# Faulty Folic Acid Assumptions: Prenatal Supplements Not Always a Good Idea

**Published:** 2006-10

**Authors:** Ernie Hood

Dietary folic acid supplementation in women of childbearing age has been a major public health success story, reducing the incidence of neural tube defects (NTDs) by an estimated 50–70%. The CDC currently recommends that all women of childbearing age eat a diet high in folic acid or take a daily multivitamin with 0.4 mg of folic acid each day, with higher intake from before conception through the first three months of pregnancy. In light of a new analysis of NTDs and folate pathway genes, however, that blanket recommendation may need to be fine-tuned **[*EHP* 114:1547–1552; Boyles et al.]**.

If these results are confirmed, it appears there may be a subgroup of women in whom folic acid supplementation is actually positively associated with the formation of NTDs in their offspring—a startling finding the authors acknowledge to be counterintuitive. Counterintuitive or not, the study may have uncovered individuals susceptible to adverse outcomes stemming from overactivity in the folate metabolism pathway during a critical stage of embryonic neurodevelopment.

The researchers analyzed the genomes of 304 families where at least one individual had an NTD. They focused their analysis on 28 single-nucleotide polymorphisms (SNPs) in 11 genes known to be involved in the folate metabolism pathway, and stratified the genomic results by potential gene–gene interactions and by whether the mothers had taken folate-containing nutritional supplements prior to conception.

The results showed that certain SNPs in the betaine-homocysteine methyltransferase (*BHMT* ) gene were significantly associated with NTDs, and that the significance was strongest with mothers who took folate supplements before conception. The group also found an associative gene–gene interaction: significance increased in the *BHMT* rs3733890 SNP when the data were stratified by preferential transmission of one particular *MTHFR* allele from parent to offspring. However, *MTHFR*, the most widely studied gene implicated in NTD research, was not found to be a significant single risk factor.

The authors speculate that the stratification method they employed may have inadvertently grouped families by one or more unidentified cofactors correlated with folate supplementation, or that the *BHMT* polymorphism may create a genetic variant that promotes overactivity of the folate metabolic cycle, given the high folate levels achieved during supplementation. This overactivity may inappropriately silence growth factors necessary for proper neural tube closure. Whether this potentially important polymorphic anomaly is grounded in analytic methodology or in actual biology, further research is clearly indicated.

NTDs are known to be complex multifactorial disorders arising from an array of genetic and environmental interactions. In their various physiologic manifestations, they are either profoundly disabling or fatal. Although the preventive effect of folic acid supplementation is undeniable, its protective mechanism of action is still poorly understood. And if the results of this study are replicated and confirmed, the public health directives concerning folic acid supplementation may need to be revised.

